# Prenatal diagnosis, care and management in Africa: bibliometric analysis

**DOI:** 10.11604/pamj.2018.29.146.11307

**Published:** 2018-03-06

**Authors:** Brice Poreau

**Affiliations:** 1 CHU Grenoble, 38000 - Grenoble, France

**Keywords:** Prenatal diagnosis, fetal alcohol syndrome, HIV, Africa, bibliometrics

## Abstract

Prenatal diagnosis, care and management are involved in mortality and morbidity of every country. A high prevalence is estimated in Africa. We use bibliometrics and mapping tools to explore the area studies and countries involved in scientific research on prenatal diagnosis, care and management in Africa. We used two databases: Web of Science and Pubmed. We extracted sets of data as publication years, organizations, funding agencies, countries from Web of Science core collection database and Medical Subject Headings from Pubmed database. We mapped the data using VOSviewer. We performed keyword analysis. We accessed 463 articles published between 1956 and 2015 in Web of Science Core collection Database and 3372 from Pubmed database. The majority of which were after 2004. The main countries involved in research on prenatal field in Africa were the USA, the United Kingdom, France and South Africa. Two main keywords are relevant: fetal alcohol syndrome and HIV. Prenatal diagnosis, care and management are leaded by South Africa. Some new countries are merging such as Rwanda. The main fields are fetal alcohol syndrome and HIV. It is funded by NIH but also Cape Town University.

## Introduction

Maternal and child survival is one of the main goals for the World Health Organization [1, 2]. The former Millennium Developmental Goals [3] and the new Sustainable Development Goals include clearly maternal and child survival by reducing under-five mortality for example. Nevertheless, in order to achieve this goal, physicians have to take into account the prenatal medicine. Prenatal medicine implies prenatal diagnosis, prenatal care and management in order to reduce maternal and child morbidity and mortality. Africa has one the highest rate of paramaternal and child mortality [3, 4]. Understanding the main strengths and weaknesses is needed to achieve the goal of reduction of maternal and child mortality and morbidity [3, 4]. The aim of this article is to analyze scientific publications on prenatal diagnosis, care and management in Africa, to determine the links between the countries involved and to highlight the main area studies and the forgotten topics.

## Methods

We used previously described methods [5-7]. Briefly, we accessed through two databases: the Science Citation Index-Expanded (SCIE) database Core collection, from the Web of Science (WOS) platform Thomson Reuters and Pubmed database. Concerning the WOS database, in the advanced search from WOS, we obtained the articles using this formula: TS = (Prenatal and Africa) for the period 1956-2015. We verified each record to ensure its relevance. And we verified the author’s affiliations. There were no restrictions regarding the document types. Then, we performed the “analysis results” function of WOS. We extracted: countries, funding agencies, organizations, publication years and Web of Science categories. In order to analyze the Web of Science categories, we exported the date into a file “analyze.txt”. This file can be read by the program wc10.exe. It generated map-files for VOS viewer [8-10]. These analyses were to compare with the search TS = Prenatal in WOS database, not restricted to Africa. We added search from Pubmed. We obtained publications using this formula: (Prenatal AND Africa) AND ("1950"[Date - Publication]: "2015/12/31"[Date - Publication]). We extracted publication years. We extracted data with MEDLINE file. We then analyzed Medical Subject Headings (MeSH) and we generated VOS-viewer diagram as described [8-10]. Finally, we performed several keyword researches to get the main relevant topics [5].

## Results

**Figure 1:**
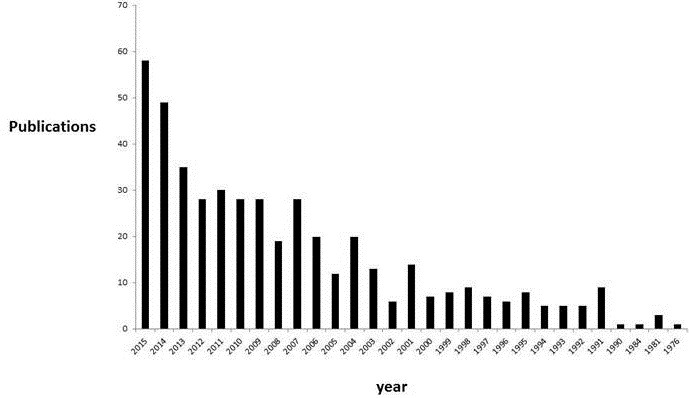
Publications (n = 463, WOS database) per year from 1956 to 2015

**Figure 2:**
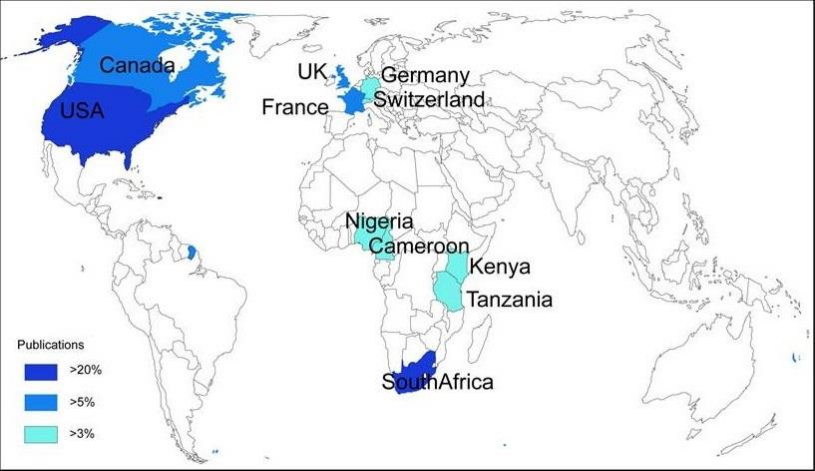
Countries involved in prenatal research in Africa according to the percentage of publications in WOS database; the United States of America (USA) and South Africa are the leaders

**Figure 3:**
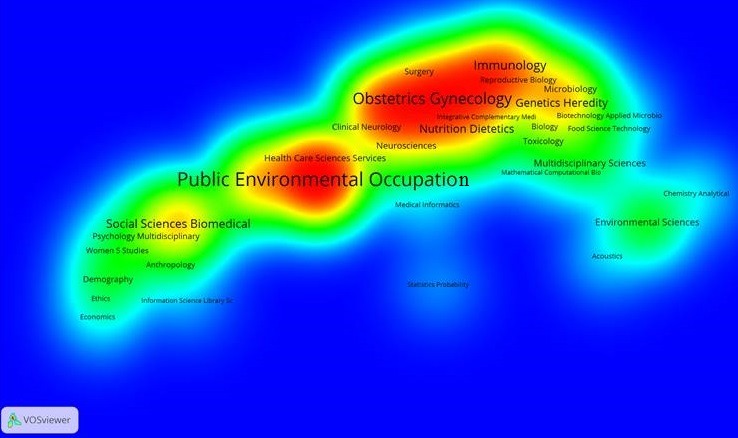
Web of science categories for the search “prenatal and Africa”; the main categories are obstetrics gynecology and public environmental health

**Figure 4:**
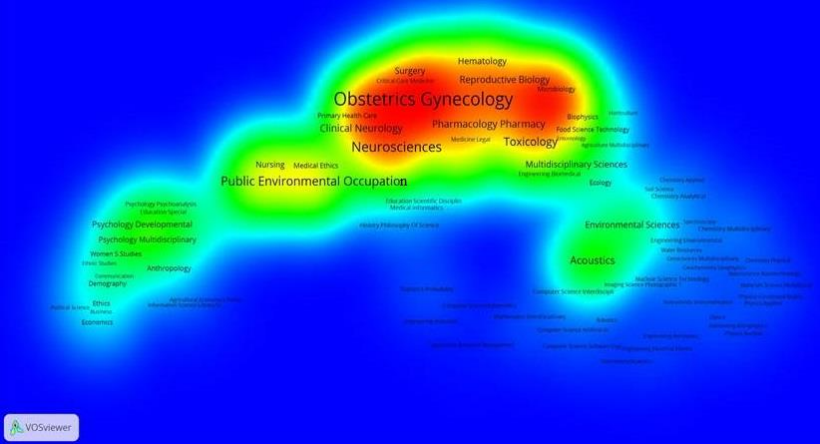
Web of science categories for the search “prenatal”; the main category is obstetrics gynecology

**Figure 5:**
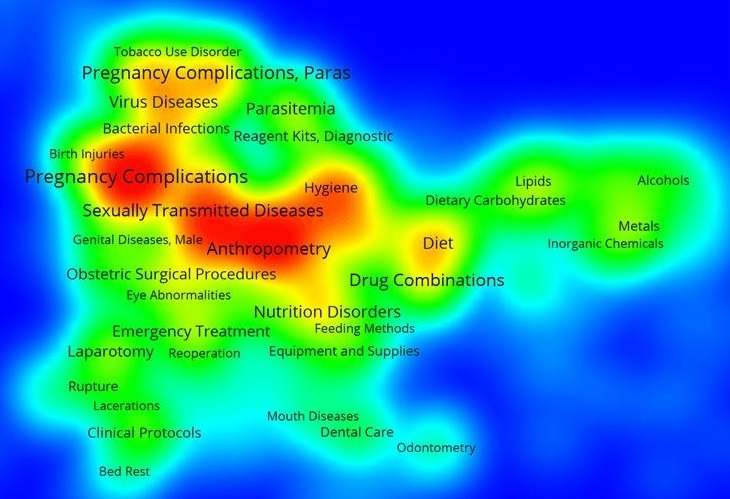
Medical Subject Headings (MeSH) of the search “prenatal and Africa” in Pubmed database; the main MeSH are pregnancy complications, sexually transmitted diseases and anthropometry

Using WOS core collection database, we obtained 463 records. More of the records were after 2004 (Figure 1). The main research funding agencies were Bill and Melinda Gates Foundation, the NIH and the University of Cape Town (South Africa). The four most represented institutions were the University of Cape-Town (South Africa) (12%), The University of Witwatersrand (South Africa) (7%), the University of California (USA) (6%) and Harvard University (USA) (6%). The main countries involved were the United States of America (42.7%) and South Africa (27.6%). We presented the countries involved according to the percentage of publications found in WOS database (Figure 2). We used VOSviewer to map the Web of Science Categories (Figure 3). The most important categories were obstetrics gynecology and public environmental health. We compare it to the map of the search without restraining to Africa (Figure 4). The most important category was obstetrics and gynecology. We then compared the data from WOS database with another database: Pubmed. We extracted the Medical Subject Headings (MeSH) from the search “Prenatal and Africa” between 1950 and 2015. 3372 articles were found. We mapped with VOSviewer the MeSH (Figure 5). Finally, keyword analysis revealed two main diseases: fetal alcohol syndrome and HIV.

## Discussion

Most articles were published after 2004 according to the analysis of two databases Pubmed and WOS. This is in accordance with indexation and publication rates of previous studies [5-7]. On the one hand, the increase of publications after 2006 is concordant with the general increase. On the other hand, this increase is concordant with new international policies on healthcare in Africa [1-3].

South Africa is the main African country involved in prenatal diagnosis, care and management. This is a confirmation of previous general studies on the leading role of South Africa in the continent [5]. In Western Africa, Nigeria and Cameroon are involved in research on prenatal care as well as Kenya and Tanzania in Eastern Africa (Figure 2). Nevertheless, the involvement of these countries is less important than South Africa. European and American countries are also involved in prenatal care. This point is clearly concordant with others previous studies on general public health [5-7].

Keyword analysis, Web of Science Category analysis and MeSH analysis reveal two main topics: fetal alcohol syndrome and HIV. In fact, public environmental health (Figure 3) is one of the two main WOS categories for Africa whereas it is not the case for prenatal bibliometric research in general (Figure 4). The topic of fetal alcohol syndrome is therefore really relevant for African bibliometric prenatal analysis. The same thing occurs with sexually transmitted diseases (Figure 5) and the topic of HIV.

Fetal alcohol syndrome or fetal alcohol spectrum disorders (FASD) are a range of disabilities due to materno-fetal alcohol exposure [11]. Main features are facial characteristics such as smooth philtrum, short palpebral fissures and thin vermillion border associated with growth retardation and central nervous system involvement. FASD can be detected during pregnancy [11, 12]. Different risk factors are associated with FASD such as age, married or not married for example [11]. Prevention and prenatal diagnosis, care and management are a burning issue for many African countries. South Africa is involved in the fight against FASD. In fact, South Africa has one the highest rate of FASD in the world: from 29 to 290 per 1000 live births [13, 14]. Nevertheless, other African countries are aware of this topic such as Ghana for example [15]. This country of Western Africa is aware of the main consequences of FASD. For African countries, some cultural arguments are given, especially in South Africa, to attempt to explain the high rates of FASD [16]. National health policies are needed to improve prenatal diagnosis of FASD and to improve antenatal education [17].

The second main topic is HIV (Figure 5). Old studies were performed [18]. An article of 1991 explained the necessity of prevent HIV transmission in Rwanda [18]. Nevertheless, the topic remains a burning issue [19]. In this study, we notice that one topic is underestimated: genetic disorders. In fact, such disorders can be detected during pregnancy by ultrasound examination when dysmorphic features are observed. Prenatal diagnosis, management and care are really important in genetic diseases. Moreover new technologies can allow us to perform quick diagnosis in certain case. South African remains the leader in Africa in that field. The WOS core collection and Pubmed were used to perform our study. Publications from African countries could be underestimated. It could therefore reinforce our results.

## Conclusion

In conclusion, prenatal diagnosis, care and management are leaded by South Africa. Moreover, some new countries are merging such as Rwanda. The main fields are fetal alcohol syndrome and HIV, funded by NIH (USA) but also Cape Town University (South Africa). Coordination of national health policies are required to improve prenatal diagnosis, care and management.

## Competing interests

The authors declare no competing interest.

## References

[1] WHO, UNICEF, UNFPA (2014). The World Bank, United Nations Population Division. Trends in Maternal Mortality: 1990 to 2013.

[2] UN Inter-agency Group for Child Mortality Estimation (2013). Levels and Trends in Child Mortality: Report 2013.

[3] United Nations (2015). The Millennium Development Goals Report.

[4] De Bernis L, Kinney MV, Stones W, Ten Hoope-Bender P, Vivio D, Leisher SH, Bhutta ZA, Gülmezoglu M, Mathai M, Belizán JM, Franco L, McDougall L, Zeitlin J, Malata A, Dickson KE, Lawn JE (2016). Lancet Ending Preventable, Stillbirths Series study group, Lancet Ending Preventable Stillbirths Series Advisory Group. Stillbirths: ending preventable deaths by 2030. Lancet.

[5] Poreau B (2014). Mapping Rwanda public health research (1975-2014). Afr Health Sci.

[6] Poreau B (2015). Mapping South African public health research (1975-2014). S Afr Med J.

[7] Poreau B (2016). Progressive and self-limiting neurodegenerative disorders in Africa: a new prominent field of research led by South Africa but without strong health policy. Pan Afr Med J.

[8] Leydesdorff L, Rotolo D, Rafols I (2012). Bibliometric perspectives on medical innovation using the Medical Subject Headings of PubMed. Journal of the American Society for Information Science and Technology.

[9] Van Eck NJ, Waltman L (2010). Software survey: VOSviewer, a computer program for bibliometric mapping. Scientometrics.

[10] Leydesdorff L, Carley S, Rafols I (2013). Global maps of science based on the new Web-of-Science Categories. Scientometrics.

[11] Riley E, Infante MA, Warren K (2011). Fetal alcohol spectrum disorders: an overview. Neuropsychol Rev.

[12] Taylor P, Jacobson S, Van der Kouwe A, Molteno C, Chen G, Wintermark P, Alhamud A, Jacobson J, Meintjes E (2015). A DTI-Based tractography study of effects on brain structure associated with prenatal alcohol exposure in newborns. Hum Brain Mapp.

[13] Olivier L, Curfs LM, Viljoen DL (2016). Fetal alcohol spectrum disorders: Prevalence rates in South Africa. S Afr Med J.

[14] May PA, Gossage JP, Marais AS, Adnams CM, Hoyme HE, Jones KL, Robinson LK, Khaole NC, Snell C, Kalberg WO, Hendricks L, Brooke L, Stellavato C, Viljoen DL (2007). The epidemiology of fetal alcohol syndrome and partial FAS in a South African community. Drug Alcohol Depend.

[15] Badoe EV (2014). Fetal Alcohol Syndrome In Ghana: Case Series Report From Korle Bu Teaching Hospital, Accra. West Afr J Med.

[16] Eaton LA, Pitpitan EV, Kalichman SC, Sikkema KJ, Skinner D, Watt MH, Pieterse D, Cain DN (2014). Beliefs about fetal alcohol spectrum disorder among men and women at alcohol serving establishments in South Africa. Am J Drug Alcohol Abuse.

[17] Olivier L, Hons BA, Urban M, Paeds FC, Chersich M, Temmerman M, Viljoen D (2013). Burden of fetal alcohol syndrome in a rural West Coast area of South Africa. S Afr Med J.

[18] Allen S, Lindan C, Serufilira A, Van de Perre P, Rundle AC, Nsengumuremyi F, Carael M, Schwalbe J, Hulley S (1991). Human Immunodeficiency Virus Infection in Urban Rwanda: Demographic and Behavioral Correlates in a Representative Sample of Childbearing Women. JAMA.

[19] Lancaster KE, Kwok C, Rinaldi A, Byamugisha J, Magwali T, Nyamapfeni P, Salata RA, Morrison CS (2015). Incident pregnancy and pregnancy outcomes among HIV-infected women in Uganda and Zimbabwe. Int J Gynaecol Obstet.

